# The Ethics of Digital Well-Being: A Thematic Review

**DOI:** 10.1007/s11948-020-00175-8

**Published:** 2020-01-13

**Authors:** Christopher Burr, Mariarosaria Taddeo, Luciano Floridi

**Affiliations:** 1 Oxford Internet Institute, University of Oxford, 1 St Giles, Oxford, OX1 3JS UK; 2 The Alan Turing Institute, 96 Euston Road, London, NW1 2DB UK

**Keywords:** Artificial intelligence, Digital well-being, Ethics of technology, Positive computing, Self-determination

## Abstract

This article presents the first thematic review of the literature on the ethical issues concerning digital well-being. The term ‘digital well-being’ is used to refer to the impact of digital technologies on what it means to live a life that is *good for* a human being. The review explores the existing literature on the ethics of digital well-being, with the goal of mapping the current debate and identifying open questions for future research. The review identifies major issues related to several key social domains: healthcare, education, governance and social development, and media and entertainment. It also highlights three broader themes: positive computing, personalised human–computer interaction, and autonomy and self-determination. The review argues that three themes will be central to ongoing discussions and research by showing how they can be used to identify open questions related to the ethics of digital well-being.

## Introduction

The expression ‘digital well-being’ refers in this article to the impact of digital technologies on what it means to live a life that is *good for* a human being in an information society (Floridi 2014a). The rapid deployment of digital technologies and their uptake by society has modified our relationships to ourselves, each other, and our environment. As a result, our individual and social well-being is now intimately connected with the state of our information environment and the digital technologies that mediate our interaction with it, which poses pressing ethical questions concerning the impact of digital technologies on our well-being that need to be addressed (Floridi 2014b). This is neatly captured in a report by the British Academy and Royal Society (2017), which placed the *promotion of human flourishing* as the overarching principle for the development of systems of data governance—an approach that is also noted in many additional reports and articles (e.g. IEEE 2017).

Some have argued that digital technologies will usher in a new era of increased productivity and help reduce social inequality by enabling better access to currently strained services, such as healthcare (Khoury and Ioannidis 2014; Schwab 2017; World Economic Forum 2018). Others have focused on how digital technologies can be used to promote well-being and further human potential by leveraging insights from the behavioural and cognitive sciences regarding human motivation and engagement (Calvo and Peters 2014; Desmet and Pohlmeyer 2013; Peters et al. 2018). However positive opportunities are counterbalanced by concerns, including whether the growing rise of mental health issues in adolescents (e.g. depression, anxiety) can be attributed to technologies such as social media (Twenge et al. 2018, Orben and Przybylski 2019) and a need for understanding the extent of disruption to labour markets and related well-being issues, which will be caused by increased automation (Frey and Osborne 2017). Such considerations are representative of a wider, ongoing discussion about the ethical issues associated with digital well-being, which has now reached a point whereby it is important to reflect on common themes that have emerged.

This article contributes to the debate on digital well-being by offering a comprehensive analysis of the relevant literature, focusing on the ethical implications of digital technologies on individuals’ and societal well-being. Our goal is to contribute to the debate and discussions referred to above, by mapping the key issues and identifying open questions for future research on digital well-being. The section ‘Social Domains: A Review of Key Issues’ provides a review of the literature, organised according to the key social domains represented in the records. The section ‘Three General Themes: Positive Computing, Personalised Human–Computer Interaction, and Autonomy and Self-Determination’ offers a more critical perspective on the reviewed literature, drawing out general themes that emerge across domains that help to identify key challenges that require further research and discussion. We conclude the article by outlining open questions and summarising its key findings. The appendix contains the methodology used for the literature review, further explains the choice of social domains and general themes, and notes key limitations.

## Social Domains: A Review of Key Issues

The review identified the following key social domains: health and healthcare, education and employment, governance and social development, and media and entertainment. The social domains were identified largely on the basis of the literature by following the emphasis of the records—in some cases the emphasis was explicit. For instance, ‘health and healthcare’ was frequently identified as a specific domain for the intended use or application of relevant technologies, but also included bioethical considerations as well (Earp et al. 2014; Klein et al. 2015; Krutzinna 2016). However, in other instances, the domains were selected for the purpose of organising the literature and drawing together closely related areas.

Invariably, and as a result of this choice, there is some overlap between the four domains. For example, the benefits of artificial intelligence (AI) and virtual reality (VR) have been explored within health and healthcare, education, and media and entertainment. However, organising the review around four domains enables a sufficiently high-level perspective to extract common themes that could provide practical utility for further discussion.

### Health and Healthcare

Physical and mental health are core components of individual well-being, and access to and the adequate provision of healthcare is vital to social well-being. The records which explore the impact and role of digital technologies in health and healthcare focus on several core themes: technology’s impact on our conceptual understanding of ‘health’ and ‘healthcare’; ethical challenges surrounding topics such as data privacy and patient autonomy; and additional concerns posed by new technologies (e.g. robotics or AI) that include questions such as ‘Who is accountable for healthcare?’, ‘How can we ensure the intelligibility of automated decisions?’, and ‘How do we ensure equal access to digital health services?’.

In healthcare, well-being is typically conceived as an individual’s quality of life (QoL), and many studies explore how digital technologies could potentially improve QoL (Feng et al. 2018; Khayal and Farid 2017; Kibel and Vanstone 2017).^1^ In some cases, the concept itself is expanded to accommodate more than just the physical or mental health of the individual. For example, in discussing their smart living concept, Keijzer-Broers et al. (2016, p. 3462) note that QoL “emphasizes a safe home environment, good health conditions and social cohesion of the individual”. The expansion of the concept of health (and related variants) as a result of technological innovations (e.g. smart tracking) has been noted by a number of sociologists and political scientists (Kickbusch 2006), including defenders of the ‘biopsychosocial model’ of health (Engel 1977). However, the emphasis on how digital technologies *empower* individuals to look after their own health is more notable than the expanded understanding of what constitutes ‘health’ and ‘QoL’ (Chen 2011; Khudair and Aloshan 2015; Leroy et al. 2014). In some cases, this empowerment has been highlighted in connection with the *enhancement of human capabilities* (Earp et al. 2014; Klein et al. 2015), and in relation to the promotion of an individual’s *self*-*determination* (Bennett et al. 2017; Taddeo and Floridi 2018; Thieme et al. 2015). This approach, influenced by both moral philosophy and public health research, suggests a shift to a more patient-centric understanding of healthcare, influenced by the empowering effects of digital technologies that help users monitor and track their physical and mental health (Amor and James 2015). Such a shift may help address some of the challenges facing national healthcare systems (e.g. ageing population), it also brings a new range of ethical risks. Two of these issues are well-known: *privacy* and *autonomy*.

Data privacy was an important concept for many of the social domains but has a specific importance in health and healthcare due to the sensitive nature of the data involved. The ethical risks surrounding use of personal and sensitive data are widely addressed (Ahn 2011; Freitas et al. 2017; Lehavot et al. 2012; Sinche et al. 2017; Soraghan et al. 2015). They range from the risks that the exposure of health-related information may pose for an individual’s well-being due to real and perceived stigmatization (Mittelstadt 2017a), to concerns about the inference of sensitive information from seemingly benign (and in some cases public) datasets (Horvitz and Mulligan 2015). The risks are most prominent in relation to smart home technologies, either in a patient’s own home or in a care home setting, where expectations of privacy are often highest (Feng et al. 2018; Margot-Cattin and Nygård 2009; Mulvenna et al. 2017; Palm 2013; Tyrväinen et al. 2018).

Autonomy, like privacy, is another theme that is widely addressed but has specific relevance in health and healthcare. A common concern—across disciplines such as medicine, philosophy, and robotics and engineering—is the tension between ensuring the safety of patients, possibly through interventions, and respecting their right to autonomy (Bennett et al. 2017; Mahoney et al. 2007; Margot-Cattin and Nygård 2009; Sharkey and Sharkey 2012; Van Hooren et al. 2007). However, several articles also warn against the blanket prescription of decontextualized ethical concepts or principles in lieu of a careful examination of how the nature of certain illnesses alter their application. A noteworthy case is the challenge of using assistive technologies for patients suffering with some form of dementia. When it comes to implementing assistive technologies (e.g., monitoring systems), as Margot-Cattin and Nygård (2009) note, people affected by dementia may not always express valid informed consent. Therefore, ensuring the safety of patients, while also respecting their autonomy, is beset with ethical issues. The simple technology system they suggest—a room access control system for patients and caregivers that could be updated with modern IoT-enabled devices—was designed to balance considerations of safety and autonomy, and provides a good example of how digital technologies alter the way one may approach value-laden concepts such as ‘autonomy’ when contextualised to a specific setting.

Going beyond the concepts of privacy and autonomy, Palm (2013) notes that, although assistive technology is often marketed as a form of care that promotes an individual’s independence or empowerment, the deployment of assistive technologies can often lead to a *transfer of care* from specialized institutions into care recipients’ homes, which in turn raises distinct legal, social, and ethical issues that are most prominent in the domain of healthcare. Within this broader issue of *transfer of care*, three additional issues emerge: *accountability*, *intelligibility*, and *accessibility.*^2^

In terms of *accountability*, several articles argue that the growing development of assistive technologies for domestic use are placing excessive burdens on informal caregivers (e.g., parents or family members) by distancing patients from professional healthcare providers and possibly decreasing the accountability of national health services (Hampshire et al. 2016; Palm 2013). This concern is closely intertwined with the second issue of *intelligibility*.

Consider how providing users with information pertaining to their health and well-being may raise anxiety originating from a misunderstanding of the information. This concern is raised particularly clearly in Hall et al. (2017), with a review of the intelligibility (and transparency) of online direct-to-consumer genetic testing services offered in the UK. As the authors note, many of these online services are marketed as online tools for enabling individuals to “make more informed decisions about their health, wellness and lifestyle” (Hall et al. 2017, p. 908). Using the good practice principles developed by the UK Human Genetics Commission (2010), they found that most of the services reviewed failed to offer some form of supplementary support services to help users “better understand or cope with the implications of test results” (Hall et al. 2017, p. 908). In a traditional patient-doctor relationship, this supplementary support is a core duty of the primary caregiver (e.g., alleviating anxiety by reporting information pertaining to an uncertain diagnosis in an appropriate manner). The primary caregiver has some degree of accountability not only for the treatment of the patient, but also their understanding of the treatment. However, this same relationship is not easily reproduced or maintained in the case of digital technologies for healthcare, sometimes referred to as electronic health (eHealth) technologies.

Finally, a number of articles raise the issue of whether eHealth technologies will be *accessible* to those who need them most. A variety of barriers to accessibility were discussed. They include poor interface design that impedes segments of the population, such as elderly patients, from accessing a particular service (Sánchez et al. 2015); overly complex presentation of information that prevents users from making sense or practical use of the recommendations (Rughiniş et al. 2015); and prohibitive costs associated with the development of the relevant technology (Sharkey and Sharkey 2012). Therefore, it is promising to see advanced technologies, such as assistive robotics or medical-grade monitoring devices, being targeted towards consumers, hopefully with the intention of improving accessibility.^3^ Increasing accessibility to healthcare through digital technologies could improve overall public health outcomes, but in doing so it is vital that accountability, intelligibility, privacy, and autonomy are also considered to avoid the associated issues raised above.

In 2005, the WHO proposed a resolution for member states to establish national strategies for implementing eHealth solutions, which they defined as “the cost-effective and secure use of information and communication technologies in support of health and health-related fields” (World Health Organisation 2005). Over a decade later, eHealth remains a key focus for policy makers. For example, the European Commission (2018) recently stated that digital technologies are necessary to address challenges such as a growing and ageing population, health workforce shortages, and the rising burden of preventable non-communicable diseases. Many of the articles in our review echo similar points, with a significant number of contributions focusing on how assistive technologies could support the independence of elderly individuals in their own home and support the challenges of residents in care homes (Asghar et al. 2015; Bennett et al. 2017; Bryant et al. 2017; Dasgupta et al. 2016; Devillier 2017; Mahoney et al. 2007; Margot-Cattin and Nygård 2009; Misselhorn et al. 2013; Mulvenna et al. 2013; Palm 2013; Reis et al. 2016; Sharkey and Sharkey 2012; Silva et al. 2015).

Digital technologies will continue to shape the development of medical research and practice in the near future. For instance, innovations in eHealth technologies enable new streams of data that can enhance a patient’s capabilities or help mitigate problems such as medication non-adherence (Dasgupta et al. 2016; Toboso 2011). In addition, machine learning (ML) technologies are offering more reliable and efficient ways to diagnose illnesses such as Alzheimer’s (Ding et al. 2018), and developments in virtual reality/augmented reality (VR/AR) technology and brain-computer interfaces are providing new research avenues for physical rehabilitation (Folgieri and Lucchiari 2017), while also offering novel treatment methods in cognitive behavioural therapy (Pot-Kolder et al. 2018). At the same time, some articles argue that digital technologies may also cause harm to user’s mental health or, possibly, contribute to behavioural addiction (Grubbs et al. 2018; Szablewicz 2010).

The use of digital technologies in healthcare does not stop with ML and robotics, it also includes social media in a clinical setting. Employing well-known principles from bioethics (Beauchamp and Childress 2013), Lehavot et al. (2012) present a thorough discussion of the ethical considerations that clinicians face, using a case study of whether to intervene following detection of suicidal posts by patients on social media.^4^ They expose the underlying tension that can emerge between the imperatives of ‘do good’ (beneficience) and ‘do no harm’ (non maleficience). For example, they note that self-disclosure of suicidal thoughts can be therapeutic for patients. As such, the benefits of automated detection of ‘at risk patients’ by machine learning (ML) algorithms may be accompanied by a high-rate of false positives due to the inability of an ML algorithm to differentiate adequately between whether a disclosure is therapeutic in nature or indicative of self-harm. Therefore, inappropriate clinical intervention, arising from an inaccurate interpretation of the individual’s online behaviour, could cause unintended harm by failing to respect a patient’s perceived boundaries of privacy and autonomy.

Nevertheless, the opportunities for using social media in healthcare are numerous. They range from enhanced capabilities for self-care, thanks to increased personalisation of information in online settings such as social media (Hamm et al. 2013), to population-scale insights about healthcare that can inform resource allocation at the policy level (Althoff 2017; Eichstaedt et al. 2015; Khoury and Ioannidis 2014). It is worth stressing that in the medium and long term, this approach may lead to favour an over-reliance on datasets derived from social media and, therefore, create too wide a distance between researchers and the population under study (Althoff 2017)—another form of the transfer of care (i.e. accountability for the well-being of research participants).

### Education and Employment

While the ethical issues posed by the use of digital technologies in education and employment can be separated—for example, the use of AI within a school for monitoring and shaping a child’s behaviour raises ethical concerns regarding consent that are distinct from those raised by the use of AI to monitor and shape an adult employee’s behaviour—there are a couple of reasons to treat them as composite parts of one domain for the purpose of the review. First, ethical concerns about the use of automated monitoring and surveillance devices for the purpose of productivity and well-being apply to both the classroom and the workplace (e.g. mental health concerns about the possible risk of increased stress and anxiety) (O’Donnell 2015; Skinner et al. 2018). Second, technological developments are changing the nature of work, requiring a shift in terms of educational curricula to focus more on digital literacy, and also requiring employees to continuously adapt to a shifting landscape by learning new skills or refining existing ones (see World Economic Forum 2018; Frank et al. 2019). Therefore, in terms of digital well-being, it is helpful to treat education and employment as closely connected.

The Future of Jobs report by the World Economic Forum addresses how technological drivers such as high-speed mobile internet, AI, big data analytics, and cloud computing, are transforming global labour markets (World Economic Forum 2018). Introducing the report, Klaus Schwab notes that “[t]he inherent opportunities for economic prosperity, societal progress and individual flourishing […] depend crucially on the ability of all concerned stakeholders to instigate reform in education and training systems, labour market policies, business approaches to developing skills, employment arrangements and existing social contracts” (World Economic Forum 2018, p. v). A number of articles address issues related to these points. For example, Pedaste and Leijen (2018) provide a brief discussion of how a variety of digital technologies, including VR/AR, could support lifelong learning, self-fulfilment and openness to new opportunities. Karime et al. (2012) offer tentative evidence pertaining to whether interactive video game-based learning could improve certain cognitive skills (e.g., memory) in students. And Baras et al. (2016) describe how smartphones could automatically detect a student’s mood and help with the management of workload through increased awareness of stress and emotional understanding. However, in each of these papers, the primary focus is on the measurable impact that a digital technology has on a behavioural or psychological attribute that may only be indirectly linked to well-being (e.g., ability to pay attention). The broader ethical impact of the technology in question, or the risk of unintended consequences, is often overlooked.^5^ This can be best illustrated through a critique of some of the reviewed publications that discuss stress management in employment and education.

Several human–computer interaction studies focus on the link between stress and individual well-being (Andrushevich et al. 2017; Baras et al. 2016; Freitas et al. 2017; Garcia-Ceja et al. 2016) and propose some form of automated measurement to infer an individual’s psychological state (e.g., detecting levels of occupational stress from the accelerometer of a smartphone). Although some contributions highlight ethical issues such as privacy (Garcia-Ceja et al. 2016), there is a notable gap concerning how the process of automated measurement could itself lead to lower levels of well-being. One paper that addresses this gap, however, is a recent study that explores how the increased use of digital technologies in schools, sometimes for the purpose of employee measurement or the management of performance targets, is related to a negative impact on well-being (i.e., increased anxiety, stress, and depression) (Skinner et al. 2018). The authors of the study also note that the way digital technologies are “implemented through managerialism in schools can have a negative impact on teachers’ morale and sense of professional identity” (Skinner et al. 2018, p. 3). This suggests that, there could also be wider unintended consequences from implementing digital technologies in some employment settings, such as interfering with a teacher’s self-determination and ability to internalise important values related to their self-identity as an educator. Although some studies explore how techniques, like gamification, could be used to promote employee engagement and self-determination (Barna and Fodor 2018; Shahrestani et al. 2017), they only partially address the problems posed by attempts to quantify the well-being of teachers and students.

Caicedo et al. (2010) raise a related concern in relation to the technical need for representing or recording the measurement outcome of a well-being assessment within the system being used (e.g., level of positive emotion). The point is succinctly expressed by their reference to the phrase “what gets measured gets managed” (Caicedo et al. 2010, p. 445). It emphasises the fact that non-quantifiable features, such as individual values or professional and personal identity, can often be overlooked, simply because the technical systems used for managerial control are unable to represent them in an appropriate format (e.g. quantitatively).

### Governance and Social Development

Many national governments have become interested in the sciences of well-being and their impact on policy (Huppert and So 2013; Stiglitz et al. 2008). As a result, there is an increased interest in the use of digital technologies, such as big data and ML, to help monitor national indicators of well-being or to develop ‘smart cities’ that could improve social well-being. A key theme in the literature is the importance for policymakers to understand public attitudes toward the development and introduction of such technologies, and how these attitudes differ from other domains, such as health and healthcare, despite related concerns involving data privacy. The technological transformations in these areas raise new ethical issues.

Horvitz and Mulligan (2015), for example, note the need for a balance when pursuing socioeconomic data between their value for policy-making and the cost in terms of increased privacy risks (e.g. exposing sensitive information). They argue that privacy regulations and laws, in the United States specifically, are based on the assumption that “the semantics of data are [sic] relatively fixed and knowable and reside [sic] in isolated context” (Horvitz and Mulligan 2015, p. 253). However, developments in ML and data analytics are challenging this conception, as new insights and inferences can be derived from existing datasets. Horvitz and Mulligan (2015) suggest that governance should be based on what they call ‘use-based’ approaches (i.e., evaluating the acceptability of data collection on the basis of how it will be used and any insights derived from the data) in order to mitigate the privacy risks of so-called ‘category-jumping’ inferences, which reveal attributes or conditions that an individual may have otherwise wished to withhold.

Ethical issues connected to governance and social development also arise in relation to digital technologies used for *smart cities* (Khayal and Farid 2017; Oliveira et al. 2014). A public policy focused study performed by IPSOS and Vodafone found that, according to their sampled respondents, “[f]uture smart city technologies have a higher acceptance and are perceived as greater digitisation benefits than health innovation” (Vodafone Institute for Society and Communications 2018, p. 41).^6^ The authors of the study claim that this is because the data necessary for smart city scenarios are less sensitive than in other domains, such as healthcare, and the scenarios themselves more tangible.

In spite of the differing attitudes towards digitisation of smart cities and digitisation in healthcare, however, many articles sought to connect the two themes, suggesting a need for further conceptual research into *societal attitudes* towards digital well-being. For example, Khayal and Farid (2017) see the development of smart cities as an important factor in improving the non-biological, socioenvironmental determinants that underlie citizen well-being, which could extend healthcare by generating additional data streams that would reflect more accurately the multidimensional nature of health *and* wellbeing. Addressing the differing societal attitudes towards healthcare and social development, also highlighted in the IPSOS and Vodafone, is a necessary preliminary step to its successful realisazion.

### Media and Entertainment

‘Media and entertainment’ is perhaps the most ill-defined social domain in this review, incorporating a wide range of use-cases for novel digital technologies, highly contextual ethical risks, and a broad set of contributing disciplines, such as sociology, data science, ethics, and human–computer interaction. In all of these situations, however, there are important ethical challenges, including opportunities to explore self-understanding or to improve feelings of social relatedness, and risks involved in the ongoing use of social media or the development of technologies such as VR/AR.

Digital technologies associated with media and entertainment offer new opportunities for promoting well-being. For example, VR/AR could help widen access to public resources such as art galleries and museums, which are often viewed as intrinsic public goods (Fassbender et al. 2010). In addition, online gaming could help improve self-understanding and emotional well-being by providing an opportunity for players to engage with different narrative forms of self-expression through their in-game avatars (Fröding and Peterson 2013; Johnson et al. 2013, 2016; Kartsanis and Murzyn 2016). Developing on the latter topic of self-expression, Kartsanis and Murzyn (2016) argue that gaming offers distinct opportunities for *self*-*exploration* that are not found within more passive forms of media. They state that “[a]ssuming and exploring different perspectives in order to empathise and understand others may indicate a eudaimonic motivation for appreciation and meaning” (Kartsanis and Murzyn 2016, p. 33). This process can be empowering and possibly lead to greater self-understanding, because a game’s character can serve as a dynamic representation of the player’s ideal-self over which they have some control, but it can also help signify possible moral shortcomings or character deficiencies in the individual’s real-world self-identity through the cultivation of in-game moral decision-making (Fröding and Peterson 2013). Johnson et al. (2013) focus on the theme of empowerment, using the psychological framework of self-determination theory (Ryan and Deci 2017), to argue that online gaming can also satisfy the need for social relatedness through processes such as helping and chatting with others in order to pursue shared goals (e.g., task planning in massive multiplayer online games).

The literature also highlights important ethical risks linked to these uses of digital technologies. For example, Kahn et al. (2013) argue that social robotics could impede development of communicative virtues and moral reasoning.^7^ In addition, Grubbs et al. (2018) adopt a psychological perspective to suggest that perceived addiction to online pornography can have a negative impact on the development of one’s religious and spiritual identity. In the case of VR/AR technologies, Madary and Metzinger (2016) discuss the ethical risks associated with long-term immersion in virtual environments, leveraging insights from philosophy of cognitive science. They explore how altering the typical structure of the environment through VR/AR technology can lead to psychological and neurophysiological changes as a result of neuroplasticity. These risks are especially troubling because they affect the neural and behavioural development of children and adolescents, and it is not yet fully understood how long-term immersion in VR could impact the development of their perceptual or motor systems. Madary and Metzinger (2016, p. 10) treat VR as a potential form of “non-invasive psychological manipulation”, and argue that this gives reason for adopting bioethical principles, such as autonomy and non-maleficence, to help critically evaluate the benefits and risks of using VR. They produce a detailed set of ethical guidelines tailored to the governance of VR and intended both for researchers and the public but note that while their ethical recommendations are a starting point for ongoing discussion more empirical research is needed to understand fully the benefits and risks associated with VR.

Social media is another topic frequently discussed in the literature, and the review identified many ethical discussions about the impact that social media may have on individual and social well-being. Starting with the positive discussions, Hart (2016) adopts a sociological perspective to argue that social media provide an opportunity for individuals to engage in ‘edgework’, purposeful engagement in risky behaviour because of its seductive character and the rewards it brings (Lyng 2005). Edgework enables individuals to better understand the limits of their emotional well-being and cultivate skills of self-determination in an online setting, in a way similar to what may happen sometimes in online gaming. Khudair and Aloshan (2015) stress that social media can empower informal caregivers (e.g., parents of autistic children) by improving their feeling of social relatedness, while at the same time offering community-driven information pertinent to their situation. Finally, Toma and Hancock (2013) suggest that social media sites can be used by individuals for the purpose of self-affirmation (i.e., validating social feedback), following a negative encounter in an offline setting (e.g., bullying) or a separate online setting (e.g., cyberbullying).

In contrast to the positive discussions, Chen et al. (2017) show how information shared by users (e.g., photographs) can be used to infer information about a user’s mental state (e.g., happiness), which in turn could be misused by social media companies experimenting with the manipulation of user’s emotional states (Kramer et al. 2014), or advertisers looking to target very specific audiences (Matz et al. 2017). Valkenburg et al. (2006) discuss how social feedback could impact the development a user’s self-esteem; Verduyn et al. (2015) explore the differences between the impact of passive and active use of social media on the user’s emotional development of affective well-being; and Ahn (2011) demonstrates how a student’s sense of safety and security is affected by privacy settings on social media sites.

Vallor (2010, p. 158, emphasis added) takes a slightly different approach, one informed by work in moral philosophy, and focuses on the “technology-driven changes in the *moral character* of IT users, rather than measuring only the *psychological and social benefits* accrued or lost through such use”. She has also developed a virtue-theoretic or eudaimonic account of well-being in order to focus on ‘communicative virtues’ (patience, honesty, empathy, fidelity, reciprocity, and tolerance), which are affected by social media (Vallor 2010, 2016). Her main concern is that psychological studies of subjective well-being often ignore this impact, even as social media seems to represent a key challenge to the development of these moral virtues. This is a worthwhile concern, but at present the existing empirical literature from the psychological sciences on social media’s impact on well-being is rather fragmented and much disagreement remains about the impact of digital technologies on subjective well-being (Orben and Przybylski 2019; Twenge et al. 2018). The state of the literature is both a consequence of the methodology of the studies (see Orben and Przybylski 2019), which use differing assumptions regarding the choice of construct to measure, and of the nature of social media, which vary in scope, purpose, and demographics from site to site (Best et al. 2014).

## Three General Themes: Positive Computing, Personalised Human–Computer Interaction, and Autonomy and Self-determination

This section complements domain-specific analysis completed in the previous section by exploring three general themes that emerged in the literature in many social domains: ‘positive computing’, ‘personalised human–computer interaction’, and ‘autonomy and self-determination’. Critical analyses of these themes shed light on the opportunities and risks related to digital well-being and on open questions that require further research and discussion.

### Positive Computing

Positive computing builds on research in *positive psychology* (Seligman and Csikszentmihalyi 2000), by adopting an interdisciplinary perspective to study the individual and social factors that foster human flourishing in order to understand how to design digital interfaces that promote users’ well-being by embedding ethics more closely within the design process (Calvo and Peters 2013; Desmet and Pohlmeyer 2013). This interdisciplinarity is emphasised by Calvo and Peters (2014, chapter 3) when they acknowledge that understanding how to design digital technologies that enhance well-being requires expertise from diverse fields such as public health, bioethics, sociology, philosophy, psychology, public policy, media studies, literature, and art—areas which are all represented in our review.

Like positive psychology, positive computing acknowledges the difference between design that focuses on creating new opportunities or capabilities for promoting well-being and design that focuses simply on identifying and removing problems. In this sense, positive computing addresses “the optimal (rather than just average) end of possible human psychological functioning” (Calvo and Peters 2014, p. 14). But optimisation requires some form of measurement, and it is in this determination that a number of pressing ethical issues arise.

The measurement of well-being presupposes a theoretical framework that enumerates its constituents and also explains why they are prudentially valuable.^8^ The lack of a generally accepted framework raises an important challenge for positive designers, as it is unclear if the kind of well-being that a technology aims to bring about is *universally accepted* as bearing prudential value, or whether it applies more locally to a subset of users. For example, a design tailored to the well-being of individuals suffering from dementia (Margot-Cattin and Nygård 2009) is likely to be very different from the one of healthy and developing child (Alexandrova 2017). Similarly, Desmet and Pohlmeyer (2013) use a broad perspective to survey art, design, and media studies, and they highlight how the wide-ranging initiatives of positive computing makes value analysis between groups challenging. They propose that design goals can be divided into *design for pleasure* (i.e., to bring about a positive affect), *design for virtue* (i.e., to bring about moral goodness), and *design for personal significance* (i.e., to help an individual attain his or her goals). This distinction is a helpful heuristic that can also be useful when considering how to embed ethical reflection into the design process. However, Desmet and Pohlmeyer (2013) note that the remit of their categories is intended to cover a multitude of domains, including social relationships, and one could question whether this makes the prudential value of social relationships (or social well-being) subservient to the prudential value of *pleasure*, *virtue*, or *personal significance*.

Embedding ethics more closely into the design process of digital technologies is becoming more common (Brey 2015; Calvo and Peters 2013; Dorrestijn and Verbeek 2013; Ijsselsteijn et al. 2006; Roeser 2012; Shahriari and Shahriari 2017; Vallor 2016). For example, privacy concerns in computer or systems engineering are considered when determining whether to process data client-side or server-side (Sinche et al. 2017; Weiss et al. 2016), how to meet the technical demand of data minimisation (Tollmar et al. 2012), and whether to avoid using more privacy-invasive monitoring (e.g. video recording technology) when alternatives are feasible and elicit greater trust in users (Feng et al. 2018; Garcia-Ceja et al. 2016; Kocielnik, et al. 2013). These developments are promising, because, as Roeser (2012) notes, when designers evaluate the possible opportunities and risk associated with their technology, they make value-laden choices about which consequences are beneficial, and thus should be promoted, and which are harmful, and thus should be avoided. She argues that it is vital that the research and design process for health and well-being technologies is sensitive to the ethical assumptions and implications latent in such consequential decision choices.

In connection with this, Peters et al. (2018) leverage research from the psychological sciences and human–computer interaction to show how the impact of design should be considered across multiple spheres of experience, ranging from the experience of an individual user at adoption of the technology to the wider experience of all members of society.^9^ Each sphere of experience raises important normative questions that target a different aspect of well-being, and can be measured using different scales (e.g., the ‘life experience’ sphere raises the question “to what extent does the technology influence the user’s experience of psychological need satisfaction in their life overall?”, prompting a reflection on life projects that are broader than the individual behaviours captured in the ‘behaviour’ sphere) (Peters et al. 2018, p. 8). Their model is the most comprehensive framework for evaluating digital well-being to date, and it makes specific use of self-determination theory to understand the impact of digital technology on motivation, engagement, and well-being.

In addition to the aforementioned need to consider “multiple spheres of experience”, some articles stress the need to consider the differing ethical demands of various domains (e.g. industrial IoT, smart homes, smart cities, health care) (Markendahl et al. 2017), and propose a kind of *reflective equilibrium* between the ethical principles or guidelines and the target technology that was proposed (Mittelstadt 2017b; Vallor 2016). The motivating idea behind these suggestions is that because technological innovation is rapid and incessant, a rigid and fixed ethical framework will be ill-equipped to deal with novel demands and will quickly become unfit-for-purpose. Designers and engineers may view this iterative process as burdensome and an impediment to innovation. However, as is evident from the previous case of the direct-to-consumer genetic testing service (Hall et al. 2017, p. 916), discussed in the sub-section ‘Health and Healthcare’, it is becoming increasingly clearer that “companies which fail to innovate ethically and responsibly not only damage public trust but are also at greater commercial risk”.

Positive computing aims to enhance human capabilities. In line with this goal, some moral philosophers have proposed using digital technologies to contribute to *moral enhancement,* understood as a more literal way of embedding ethics into technology by offloading decision-making to an artificial moral agent (Giubilini and Savulescu 2018). Noting that humans are “suboptimal information processors, moral judges, and moral agents,” artificial moral advisors could promote a new form of personal *reflective equilibrium* by enabling an individual to consider aspects of their moral character which need improvement or refinement (Giubilini and Savulescu 2018, p. 170). While this kind of *technologically*-*mediated moral enhancement* may appear a somewhat futuristic proposal, the topic of moral and cognitive enhancement is an important part of positive computing (Earp et al. 2014; Klein et al. 2015). Krutzinna (2016) explores how emerging technologies, such as brain-computer interfaces, could potentially enhance our cognitive abilities and lead to greater levels of well-being, but also raise more immediate concerns about digital well-being. For instance, she argues that well-being is often deployed as a somewhat vague concept and thus imposes too few practical constraints on an individual’s decision-making. To illustrate this point, we can consider a parent who is trying to determine whether to impose constraints on a child’s social media usage on the basis that it diminishes attentional capacities and thus leads to lower levels of well-being. Because ‘well-being’ is inadequately specified, however, it can play no instrumental role over and above the considerations pertaining to the potential diminishment of the attentional capacities of a child. Her argument stresses the need for greater theoretical specificity of well-being, i.e., making explicit its constituents or determinants that are being assumed.^10^ Here, one sees a larger gap, which requires further discussion: how should one design and implement technology when faced with conceptual and normative uncertainty about the consequences of specific choices?

The need for conceptual clarity and a greater consideration of the ethical challenges is also emphasised by Floridi et al. (2018), when presenting an ethical framework that is designed to complement existing sociolegal frameworks and help evaluate the opportunities and risks for society that are associated with technologies such as AI. They note that, “[c]ompliance with the law is merely necessary (it is the least that is required), but significantly insufficient (it is not the most than can and should be done)” Floridi et al. (2018, p. 694). Ethics often demands that an agent goes over and above what is legally and technically permissible, but this does not mean that it should be viewed as a barrier to innovation. Rather, ethics has the *dual*-*advantage* of enabling “organisations to take advantage of the social value that AI enables” as well as anticipating and avoiding costly mistakes that “turn out to be socially unacceptable and hence rejected, even when legally unquestionable” (Floridi et al. 2018, p. 694). Therefore, in order to ensure that the full potential of positive computing can be realised, it is crucial to continue to develop ethical guidelines that engage diverse multi-stakeholder groups, in order to understand better what is socially acceptable.

### Personalised Human–Computer Interaction

The ubiquity of digital technologies that are equipped with sensors for monitoring user behaviour and environmental conditions, combined with advances in data management and analytics, has resulted in increased viability of personalised human–computer interaction (e.g. personalised recommendations). Personalised human–computer interaction, as a general theme, was found in many domains, such as personalised healthcare and personalised recommendations in media and entertainment. This section explores the theme of personalisation, with an emphasis on usability and accessibility, and identifies a number of opportunities and risks associated with personalisation as a design strategy for digital well-being.

Personalisation is defined as “the ability to provide contents and services tailored to individuals based on knowledge about their needs, expectations, preferences, constraints, and behaviours” (Vallée et al. 2016, p. 186). Similarly, Spanakis et al. (2014, p. 23) claim that “personalised mobile technologies based on behavioural and motivational models may prove to be valuable persuasive tools and means to foster healthier life styles and sense of wellbeing throughout an individual’s life span.” For example, personalisation may allow for more focused consideration of fine-grained notions of well-being, such as ‘maternal well-being’ (see McDaniel et al. 2012). However, many forms of personalised technology, especially those that learn about user preferences, require monitoring of relevant behavioural signals in order to measure improvement and track goal progress. Public attitude towards these techniques has been impacted negatively by recent data abuses stemming from micro targeting of advertisements on the basis of such data (PEW Research Center 2018).

Two central elements of positive design are usability and accessibility. The first element is the consideration of whether some technology is usable in an *ergonomic* sense, i.e., whether it is cumbersome to use or burdens the user by requiring an excessive level of information input. However, as Meyer and Boll (2014) note, focusing too much on one usability requirement, such as *unobtrusiveness* (e.g., designing small sensors that can be embedded into smart textiles, or designing systems that attempt to automate and outsource some part of the decision-making process), can have a negative impact on a user’s well-being if the resulting measurement outcome is less accurate than a more obtrusive alternative. At the same time, design considerations need to include considerations about *accessibility*, which here refers to whether the design is usable for all members of a population (also see section ‘Health and Healthcare’). In this vein, Toboso (2011) highlights the challenge that people with disabilities face when using certain technologies from the perspective of the human development and capabilities approach (Sen 2010). Citing the United Nations Convention on the Rights of Persons with Disabilities, Toboso (2011, p. 114) emphasizes that ‘universal design’ is “the design of products, environments, programmes and services to be usable by all people, to the greatest extent possible, without the need for adaptation or specialized design.” The goal of universal design is laudable, but as the review indicates is poorly explored in the literature at present.

Going beyond considerations of disability, a number of articles discuss how, for example, interface design often fails to consider the accessibility requirements of certain groups of individuals (e.g., elderly users), thus restricting their access to technologies that could promote their well-being (Sánchez et al. 2015; Silva et al. 2015). Some restrictions are physical, but others arise in a different way, e.g., assumptions about the levels of media literacy of users. In relation to media literacy, the notion of accessibility of information must not be construed simply as the removal of barriers, but also as the curation of information in ways that respect an individual’s abilities. As Bryant et al. (2017, p. 5) note, “[i]t is estimated that 12.6 million adults (23%) in the UK do not have the required level of aptitude in managing information, communicating, transacting, problem solving and creating. Among people aged 65+ this rises to 43% and is the group that has the lowest digital device ownership and are often retired so could lack access to technology that working people often have.”

Consideration of media literacy helps bring into focus important aspects of what one may term *epistemic accessibility*, presenting information in ways that afford users actionable and perhaps personalised insights, rather than simply burdening them with information, or raising anxiety through inappropriate delivery of sensitive information (e.g., healthcare). As one possible solution from software engineering, Mitrpanont et al. (2018) explore how a chatbot could be incorporated into a monitoring system that is designed to notify an individual about environmental information relevant to their health (e.g., air pollution or noise pollution), potentially guaranteeing the individual has a way to ensure they understand the information being presented to them.

It is understandable that designers may feel uncomfortable with some of the suggestions explored in this section, especially in light of the growing debates around paternalistic design choices (Floridi 2016), or the possible risks of polarisation stemming from increasingly personalised data streams. However, to unlock the value of personalised technology it is important to tackle these concerns head-on. As discussed in the next section, a shift in how one understands related concepts such as autonomy, capabilities, and self-determination, may help alleviate some of these issues.

### Autonomy and Self-determination

Autonomy has become an important topic in relation to the interactions between human users and digital technologies, especially persuasive technologies that seek to learn about a user’s preferences and steer their behaviour towards pre-determined goals (e.g. maximising engagement) (see Burr et al. 2018 for a discussion). Unsurprisingly, therefore, the ethical issues related to autonomy are discussed across a wide-range of records, spanning fields such as psychology, philosophy, public health, and design studies (Calvo and Peters 2013; Desmet and Pohlmeyer 2013; Hart 2016; Lehavot et al. 2012; Rughiniş et al. 2015; Taddeo 2014; Vallor 2010; Van Hooren et al. 2007). However, the approaches taken to autonomy differ widely, suggesting that particular aspects of the concept are only perspicuous when considering specific implementations of digital technology (e.g., an assistive technology for home care).

Rughiniş et al. (2015), for example, extract five dimensions of autonomy that are useful for understanding the mediating role that health and well-being apps have on the communication of information. The dimensions are (a) degree of control and involvement that the user has within the app; (b) degree of personalisation over the apps functionality; (c) degree of truthfulness and reliability related to the information presented to the user, and how this affects their decisions; (d) user’s own self-understanding regarding the goal-pursuit, and whether the app promotes or hinders a user’s awareness of their own agency; and (e) whether the app promotes some form of moral deliberation or moral values in the actions it recommends. These five dimensions help to bring some aspects of autonomy into greater focus but may also obscure others. For example, although Rughiniş et al. (2015) make room for *social relatedness* within their five dimensions, defenders of substantive-relational accounts of autonomy may argue that it requires greater emphasis as a dimension in its own right (MacKenzie 2008).

With regards to well-being, what is most important about the autonomy debate is that one keeps in mind how a *freedom to choose and to self*-*determine* is often understood as an intrinsic good or right, rather than merely a means to secure well-being. As Sen (2010, p. 18) notes: “The freedom to choose our lives can make a significant contribution to our well-being, but going beyond the perspective of well-being, the freedom itself may be seen as important. Being able to reason and choose is a significant aspect of human life. In fact, we’re are under no obligation to seek only our own well-being, and it is for us to decide what we have good reason to pursue.”

Concern for self-determination does not mean that any technologically-mediated intervention should be avoided in case it restricts a user’s freedom to choose. Indeed, several contributions refer to Sen’s capability approach to show how some technologically-mediated interventions, which could initially be misconstrued as paternalistic, can in fact be treated as autonomy-enhancing, if one views autonomy as intimately connected to an individual’s social relations and environmental affordances (Kibel and Vanstone 2017; Misselhorn et al. 2013; Taddeo 2014; Toboso 2011). What is needed is greater attention to these more developed theoretical accounts of autonomy in the development of digital technologies and the political and ethical discussions that shape their design and regulation.

When one considers accounts of autonomy that are more developed than simplistic dichotomies and tensions between autonomy and automation, specific ethical issues become clearer. For example, technologically-mediated nudges, often viewed as archetypal forms of paternalistic interventions, can sometimes be used to promote deliberative capacities (Levy 2017). In turn, they can help people avoid the problem of outsourcing decision-making to autonomous systems (Vallor 2016). This problem is typically framed from a virtue ethics perspective (Shahriari and Shahriari 2017; Vallor 2016), and these authors emphasise the importance of conscious contemplation for the cultivation of human excellence. However, even if one chooses not to adopt a virtue ethics perspective, one can still appreciate why digital technologies that seek to present users with information designed to elicit greater deliberative (perhaps moral) faculties are worthwhile.

In connection with this aspect, Rughiniş et al. (2015) argue that the ‘truthfulness’ of communicative messages requires further study, as the manner in which motivational notifications and nudges are framed could have a negative impact on an individual’s well-being. For example, the fast and frugal nature of app communication, influenced by design principles from persuasive technology that target an individual’s cognitive biases, often favours interventions on an individual’s *extrinsic motivation* (Desmet and Pohlmeyer 2013). However, as is well-known, targeting extrinsic motivators is often a short-term solution to behaviour change that bypasses the development of intrinsic motivators, the ones that promote long-term and sustainable self-determination (Ryan and Deci 2017). The importance of intrinsic motivation for sustainable well-being has also been noted by advocates of positive computing (Peters et al. 2018), and is changing how features like gamification are being used to promote self-determination and user engagement (da Silva et al. 2013; Hall et al. 2013; Shahrestani et al. 2017). There is a key opportunity here for reconceiving the debate over autonomy by situating it within a psychological framework, such as self-determination theory (SDT), which treats autonomy as one basic psychological need among others.^11^

Our analysis stressed that the apparent tension between autonomy and automation has resulted in conceptual confusion regarding a cluster of topics such as self-determination, self-understanding, and identity (both individual and social). Not all forms of artificial decision-making entail a constraint on human autonomy, but to appreciate this fact one first needs a clearer understanding of the varied nature of human–computer interaction (see Burr et al. 2018). It is promising, therefore, that these issues are being widely discussed and that more nuanced theoretical accounts of autonomy are being defined (Peters et al. 2018; Rughiniş et al. 2015). A greater awareness and conceptual understanding of these theoretical developments could lead to a better scrutiny of digital technologies (and related social policies) that impact human well-being.

These general themes are intended as a starting point for subsequent discussion. In the next section, we offer some further remarks to help guide the next stage of the discussion.

## Conclusion

In this section we outline some open questions that we hope will motivate researchers from a wide range of disciplines to engage further with the topic of digital well-being. In closing this paper, we offer some further remarks about the key findings of this review.

### Open Questions

Connecting the three themes explored in the previous section to the key issues identified by the review of the social domains allows us to identify some open questions related to the ethics of digital well-being. The examples provided in Table 1 are not intended to be an exhaustive list of open questions on ethics of digital well-being. Rather, the list provides the starting point for further research and discussion on this topic.

**Table 1:** Key issues and open questions related to the ethics of digital well-being. The table is organised according to the key social domains and general themes identified in the review

	Key issues	Positive computing	Personalised human–computer interaction	Autonomy and self-determination
Healthcare	Patient empowerment or enhancement of capabilities Privacy risks (e.g. use of sensitive data) Trade-off between safety and autonomy Transfer of care: accountability, intelligibility, accessibility	Does the incorporation of positive computing techniques into the domain of healthcare risk expanding or trivialising the concept of ‘health’ (e.g. more health is always possible)? In healthcare, how should one weight design considerations such as pleasure, virtue and personal significance when seeking to promote QoL?	Can personalised treatment be achieved while minimising the collection of sensitive data? If not, do the benefits (e.g. increased usability or accessibility) outweigh the risks (e.g. increased anxiety)? Is technologically-mediated personalised treatment the best way to improve accessibility requirements and achieve the goal of universal design?	How can empowerment or enhancement of a patient’s capabilities be achieved while ensuring that responsibility or accountability for adequate care is not transferred to informal caregivers? How should specific assistive technologies balance the often-contrasting considerations of patient safety and autonomy?
Education and employment	Changing needs of labour markets and importance of lifelong learning Automated monitoring or measurement of subjective well-being Self-understanding or identity	Should one use positive computing methods to improve student or employee engagement?	Should personalised monitoring of employee well-being be used?	How do digital technologies alter an employee’s self-understanding or identity? Should digital technologies be used to enhance our moral capacities, or does this impede on an important aspect of the virtue of moral deliberation?
Governance and social development	Societal attitudes towards trade-offs between privacy risks and potential value from population-scale datasets Greater awareness of environmental impacts on health and well-being	Could positive computing methods help promote social well-being, or are they only applicable at the individual level?	How can one protect an individual’s privacy (or feelings of privacy) while unlocking the social value of big datasets?	How can smart cities enhance the capabilities of citizens and empower communities?
Media and entertainment	Empowerment (e.g. promotion of self-understanding or emotional well-being through interactive media) Developmental concerns from new technologies (e.g. VR) Impact of social media on psychological well-being and moral character	What are the risks associated with using positive computing methods to improve engagement with media (e.g. games that are too engaging may lead to behavioural addiction)? In lieu of scientific consensus regarding the impact of social media on mental health, how should one evaluate the possible risks (as parents, as a society, or as individuals)?	Are ethical guidelines sufficient to ensure our digital footprints are not misused? Or, are stricter legal frameworks required?	How should social media platforms be designed in ways that promote feelings of social relatedness?

### Further Remarks

It is likely that different communities will concentrate on one or more of the four social domains or three themes to differing degrees. For example, psychologists will find that their expertise enables them to utilise advances in ML and social data science to develop new constructs and measures that can help delineate the scope of digital well-being.^12^ Legal scholars and ethicists will find that their expertise is needed to address questions relating to data governance, which will emerge alongside growing interest in more personalised human–computer interaction. These different priorities, however, should not suggest the need for a strict division of labour, as the issues and possible solutions are inherently interdisciplinary. In our review, the positive computing approach stands out as a noteworthy example in this regard because it utilizes a framework that clearly demonstrates the need for interdisciplinary perspectives in the ongoing development of well-being supporting digital technologies (Calvo and Peters 2014). It is to be hoped that this review will serve as a starting point for other interested parties to become involved and help to deliver on the promise of digital technologies that promote flourishing for all.

## Figures and Tables

**Fig. 1:**
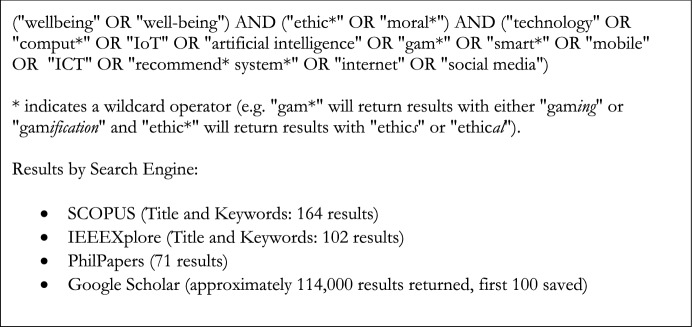
Generic form of the search query used and the results by search engine

**Fig. 2:**
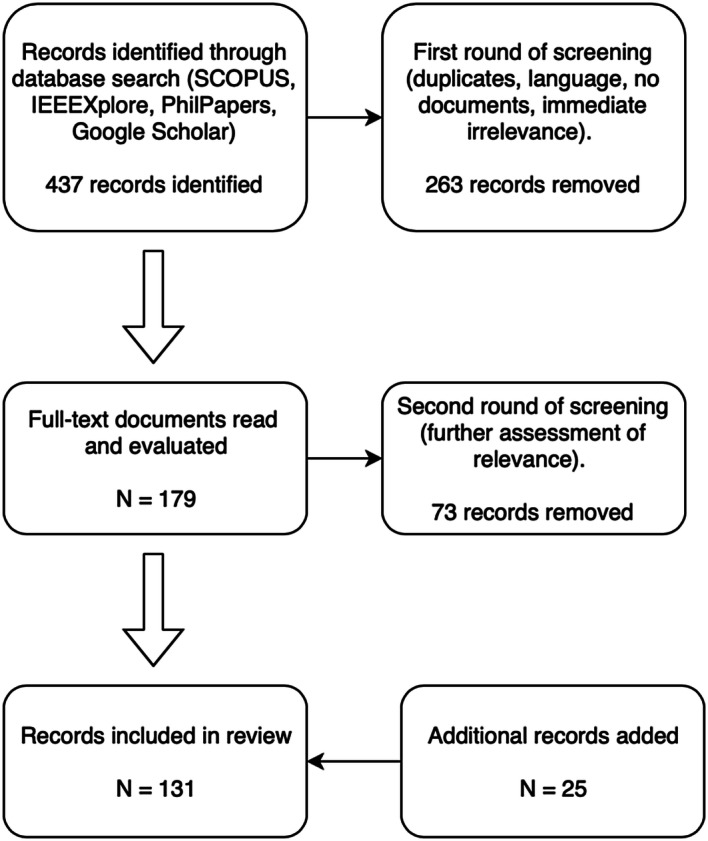
A flowchart indicating the stages of our review

## Appendix: Methodology

The analysis began by searching four databases (SCOPUS, IEEEXplore, PhilPapers, and Google Scholar). Figure 1 provides the generic form of our search query, which was adapted to the specific syntactical requirements of each search engine and run on the 25th October 2018.^13^ For SCOPUS and IEEEXplore, the search string was restricted to Title and Keywords to ensure the return of the most relevant papers. For IEEEXplore, the “AND (“ethic*” OR “moral*”)” portion of the string was omitted due to limited results with the full string, and so the filtering of papers to those dealing with ethical issues was performed manually during the second stage. No other filters or restrictions were employed (e.g. date range).

This search returned a total of 437 records, which were then screened in two stages. The first stage removed duplicate entries, records in a non-English language (see sub-section ‘limitations’), and citations for which no document could be obtained due to licensing restrictions. The titles and abstracts were then assessed for relevance based on the criteria highlighted at the start of this section. A total of 263 records were excluded at this stage. The remaining 179 records, spanning records from 2002 to 2018, were read in their entirety.

The concept of ‘well-being’ and its variants (e.g. ‘welfare’, ‘happiness’, and ‘flourishing’) are widely used (often indiscriminately), and play various roles depending on the disciplinary context. As such, one expected to find a large number of studies that were of no direct relevance to our review, but which were returned due to overly liberal use of the term ‘well-being’. In addition, the goal was not to provide a meta-analysis of empirical studies that sought only to establish whether some digital technology impacts well-being, according to some measure of well-being.^14^ Therefore, a second round of exclusion was also conducted to remove records that did not raise some ethical issue pertaining to well-being and digital technology, leaving 106 records in total from the initial search query. Finally, 25 records were also added that were known to the authors prior to carrying out the review, and which offered additional perspectives on the themes raised by the original search results. These records are identified in the bibliography using an asterisk [*]. Therefore, a total of 131 records formed the basis of our review, which is summarised in Fig. 2.

### Identifying the Social Domains and General Themes

The social domains and themes were identified *ex post* in a semi-systematic manner, rather than using a pre-defined thematic framework that restricted inclusion of relevant themes. The identification of the social domains was decided through group discussion and based on a several relevant factors: the domain-specific use-case for the respective technology, as identified by the authors (e.g. health and healthcare); the disciplinary focus of the publication; the keywords selected by the authors; and the wider themes discussed in the record (e.g. policy-related implications.

The domain of ‘health and healthcare’ represents the most significant portion of the review, with 44 records *directly concerned* with health or healthcare in some form. In contrast, ‘education and employment’ included 10 records, ‘governance and social development’ included 5 records, and ‘media and entertainment’ included 20 records. The remaining 52 records were either indirectly related to multiple domains or focused on more general issues.

A semi-systematic method was chosen due to intended goal of the review, which was to map the ethical issues and themes associated with the literature on digital well-being. Ultimately, this means that there will be a degree of author bias introduced into our decision of how to group the domains, which others may disagree upon. However, the method allowed us to focus on domains that we felt best summarised the literature from a high-level perspective, providing a useful overview that we believe has practical utility in a multi-disciplinary review. Nevertheless, this perspective led to a significant amount of overlap between the records, which subsequently led to a decision to also focus on general themes that emerged when we started our analysis of the social domains.

As the domain-oriented section of the paper shows, the three themes are not wholly representative of the literature. For example, ‘data privacy’ was also a general theme that arose across several domains, including ‘health and healthcare’ and ‘media and entertainment’, but which was not explored due to extensive work on data privacy within other communities.^15^ Instead, we selected the three themes of ‘positive computing’, ‘personalised human–computer interaction’, and ‘autonomy and self-determination’ because of their perceived novelty and importance for the further multidisciplinary and interdisciplinary study of digital well-being.

27 records were included within the theme of positive computing, in some cases because of their explicit focus on ‘positive design’; 11 records discussed themes related to personalised human–computer interaction; and 20 records discussed ethical issues related to autonomy and self-determination.

### Further Limitations

There are two further limitations of this study. First, the review was restricted to records written in English, although only 15 non-English language documents were *removed* as a result of this restriction. This means that some ethical issues or analyses of ethical issues have been overlooked, and that the review offers a limited comparative analysis of research developed in different countries and region, insofar as it included only contributions in English. This type of analysis would be useful, especially given the recent focus on exploring the global landscape of AI ethics guidelines (e.g. Jobin et al. 2019; Floridi et al. 2018). Second, by adopting a high level of abstraction for this review, ethical issues that are only made perspicuous following additional contextual specification may also have been overlooked. This is unfortunately an unavoidable trade-off between the type of scoping perspective provided by the current review and the more detailed analysis offered by an individual case-study approach.

To overcome these limitations, further research could (a) expand the literature review by including non-English records, or (b) take a case-study approach to individual domains and analyse specific ethical issues in more detail. Such tasks are left to further research, and we hope that our semi-systematic review offers an informative starting point for these valuable tasks.

## Footnotes

1 More specific variants also exist, such as ‘quality-adjusted life years’ or ‘disability-adjusted life years’ (see Hausman 2015 for a discussion).

2 These issues are merely representative and not intended to be exhaustive. For example, reliability or trustworthiness of information are also noteworthy problems that raise distinct ethical concerns.

3 Key examples here are the inclusion of an EKG sensor on the most recent iteration of the Apple Watch in the US (https://www.wired.com/story/apple-watch-series-4/), and Samsung’s announcement of a new range of robotics for assisted living (https://www.cnet.com/news/bot-care-leads-a-legion-of-new-samsung-robots-at-ces-2019/).

4 See Burr and Cristianini (2019) for an overview and discussion of automated detection of psychological traits or mental states from digital footprints (including social media).

5 One exception is O'Donnell (2015), who argues that by co-opting the practice of mindfulness, in order to help students improve their ability to pay attention or better cope with information overload, the ethical orientation of the practice itself becomes programmatic rather than pedagogical.

6 The quantitative research sampled 9005 adults aged 18–65 across nine countries.

7 Similar concerns have emerged in the popular media surrounding the growing use of voice-activated smart assistants and the impact that their use has on the development of conversational norms in children (Gonzalez 2018). These concerns have led technology manufacturers to respond by incorporating new features into their devices that can reward politeness in children, though the wider psychological impact is still poorly understood.

8 The term ‘prudential value’ is used in philosophy to separate that which is intrinsically good for a person (i.e. well-being) from that which is merely good because of its instrumental role in leading to a greater level of well-being (e.g. income, employment, social network) (see Fletcher 2016).

9 The full range of spheres includes ‘adoption’, ‘interface’, ‘task’, ‘behaviour’, ‘life’, and ‘society’. See Peters et al. (2018) for a full description of the spheres and how they relate to each other.

10 The lack of both theoretical and empirical understanding of the goals of well-being is one of the most important gaps identified in the literature (see Krutzinna 2016). This gap could result from, among other things, disagreement regarding the best way to measure a particular well-being construct, or from uncertainty about the causal relationship between the use of a digital technology (e.g., a smartphone) and a psychological effect (e.g., increased anxiety or depression). For example, there has been a wide, and yet unsettled, discussion about the impact that screen time has on subjective well-being (Gonzalez 2019; Orben and Przybylski 2019; Twenge et al. 2018). As of writing, there still appears to be much uncertainty in the scientific community regarding the scope and validity of empirical claims that underlie these discussions.

11 SDT identifies *three* basic needs (competence, autonomy, and relatedness), which must be satisfied for an individual to “experience an ongoing sense of integrity and well-being or "eudaimonia"” (Ryan and Deci 2000, pp. 74–75).

12 Because of the interdisciplinary nature of these challenges, there is also a vital role here for philosophers of science. This is clearly discussed by Alexandrova (2017) who draws attention to the mixed nature of well-being claims and presents a framework for connecting conceptual and normative research in philosophy with empirical research in the sciences of well-being.

## References

[CR1] Ahn J (2011). The effect of social network sites on adolescents’ social and academic development: Current theories and controversies. Journal of the American Society for Information Science and Technology.

[CR2] Alexandrova A (2017). A philosophy for the science of well-being.

[CR3] Althoff T (2017). Population-scale pervasive health. IEEE Pervasive Computing.

[CR4] Amor, J. D., & James, C. J. (2015). Setting the scene: Mobile and wearable technology for managing healthcare and wellbeing. In *Presented at the 2015 37th annual international conference of the IEEE engineering in medicine and biology society (EMBC)* (pp. 7752–7755). 10.1109/EMBC.2015.7320189 26738089

[CR5] Andrushevich, A., Biallas, M., Kistler, R., Eusebi, L., Ronai, J., & Valla, M. (2017). Towards smart working spaces with enhanced well-being and safety of elderly staff. In *Presented at the 2017 global internet of things summit (GIoTS)* (pp. 1–6).

[CR6] Asghar, I., Cang, S., & Yu, H. (2015). A systematic mapping study on assitive technologies for people with dementia. In *Presented at the 2015 9th international conference on software, knowledge, information management and applications (SKIMA)* (pp. 1–8).

[CR7] Baras, K., Soares, L., Paulo, N., & Barros, R. (2016). ‘Smartphine‘: Supporting students’ well-being according to their calendar and mood. In *Presented at the 2016 international multidisciplinary conference on computer and energy science (SpliTech)* (pp. 1–7).

[CR8] Barna, B., & Fodor, S. (2018). Gamification’s impact on employee engagement: Enhancing employee well-being with a cloud based gamified team-building application. In *Presented at the 2018 6th international conference on future internet of things and cloud workshops (FiCloudW)* (pp. 203–208).

[CR9] Beauchamp T, Childress J (2013). Principles of biomedical ethics.

[CR10] Bennett B, McDonald F, Beattie E, Carney T, Freckelton I, White B, Willmott L (2017). Assistive technologies for people with dementia: Ethical considerations. Bulletin of the World Health Organization.

[CR11] Best P, Manktelow R, Taylor B (2014). Online communication, social media and adolescent wellbeing: A systematic narrative review. Children and Youth Services Review.

[CR12] Brey, P. (2015). Design for the value of human well-being. In J. van den Hoven, P. Vermaas & I. van de Poel (Eds), *Handbook of ethics, values, and technological design. Sources, theory, values and application domains* (pp. 365–382). Springer [*].

[CR13] British Academy and Royal Society. (2017). Data management and use: Governance in the 21st century. https://royalsociety.org/~/media/policy/projects/data-governance/data-management-governance.pdf. Accessed February 2, 2018 [*].

[CR14] Bryant, N., Spencer, N., King, A., Crooks, P., Deakin, J., & Young, S. (2017). IoT and smart city services to support independence and wellbeing of older people. In *Presented at the 2017 25th international conference on software, telecommunications and computer networks (SoftCOM)* (pp. 1–6).

[CR15] Burr C, Cristianini N (2019). Can machines read our minds?. Minds and Machines.

[CR16] Burr, C., Cristianini, N., & Ladyman, J. (2018). An Analysis of the interaction between intelligent software agents and human users. *Minds and Machines*, *28*(4), 735–774 [*]. 10.1007/s11023-018-9479-0 PMC6404627 30930542

[CR17] Caicedo M, Mårtensson M, Roslender R (2010). Managing and measuring employee health and wellbeing: A review and critique. Journal of Accounting & Organizational Change.

[CR18] Calvo RA, Peters D (2013). Promoting psychological wellbeing: Loftier goals for new technologies [opinion]. IEEE Technology and Society Magazine.

[CR19] Calvo, R. A., & Peters, D. (2014). *Positive computing: Technology for wellbeing and human potential*. Cambridge: MIT Press [*].

[CR20] Chen H (2011). Smart health and wellbeing. IEEE Intelligent Systems.

[CR21] Chen, Y., Mark, G., Ali, S., & Ma, X. (2017). Unpacking happiness: Lessons from smartphone photography among college students. In *Presented at the 2017 IEEE international conference on healthcare informatics (ICHI)* (pp. 429–438).

[CR22] da Silva, J. P. S., Schneider, D., de Souza, J., & da Silva, M. A. (2013). A role-playing-game approach to accomplishing daily tasks to improve health. In *Presented at the proceedings of the 2013 IEEE 17th international conference on computer supported cooperative work in design (CSCWD)* (pp. 350–356).

[CR23] Dasgupta, D., Reeves, K. G., Chaudhry, B., Duarte, M., & Chawla, N. V. (2016). eSeniorCare: Technology for promoting well-being of older adults in independent living facilities. In *Presented at the 2016 IEEE international conference on healthcare informatics (ICHI)* (pp. 461–472).

[CR24] Desmet PM, Pohlmeyer AE (2013). Positive design: An introduction to design for subjective well-being. International Journal of Design.

[CR25] Devillier N (2017). Aging, well-being, and technology: From quality of life improvement to digital rights management—A French and European perspective. IEEE Communications Standards Magazine.

[CR26] Ding Y, Sohn JH, Kawczynski MG, Trivedi H, Harnish R, Jenkins NW (2018). A deep learning model to predict a diagnosis of alzheimer disease by using 18F-FDG PET of the brain. Radiology.

[CR27] Dorrestijn S, Verbeek PP (2013). Technology, wellbeing, and freedom: The legacy of utopian design. International Journal of Design.

[CR28] Earp BD, Sandberg A, Kahane G, Savulescu J (2014). When is diminishment a form of enhancement? Rethinking the enhancement debate in biomedical ethics. Frontiers in Systems Neuroscience.

[CR29] Eichstaedt JC, Schwartz HA, Kern ML, Park G, Labarthe DR, Merchant RM (2015). Psychological language on Twitter predicts county-level heart disease mortality. Psychological Science.

[CR30] Engel GL (1977). The need for a new medical model: A challenge for biomedicine. Science.

[CR31] European Commission. (2018). *Communication on enabling the digital transformation of health care in the digital single market; empowering citizens and building a healthier society.* https://ec.europa.eu/digital-single-market/en/news/communication-enabling-digital-transformation-health-and-care-digital-single-market-empowering, Date Accessed January 7, 2019 [*].

[CR32] Fassbender, E., Wade, B., Carson, D., & Lea, T. (2010). Virtual galleries: First insights into the effect of the introduction of new media technologies, in art galleries, on economic and social wellbeing in urban and remote communities of the Northern Territory of Australia. In *Presented at the 2010 16th international conference on virtual systems and multimedia* (pp. 357–360).

[CR33] Feng Y, Chang C, Ming H (2018). Engaging mobile data to improve human well-being: The ADL recognition approach. IT Professional IS.

[CR34] Fletcher G (2016). The philosophy of well-being: An introduction.

[CR35] Floridi, L. (2014a). The Fourth Revolution. Oxford: Oxford University Press [*].

[CR36] Floridi, L. (2014b). The onlife manifesto. In *The onlife manifesto* (pp. 264–13). Cham: Springer. 10.1007/978-3-319-04093-6_2 [*].

[CR37] Floridi L (2016). Tolerant paternalism: Pro-ethical design as a resolution of the dilemma of toleration. Science and Engineering Ethics.

[CR38] Floridi L (2018). Soft ethics and the governance of the digital. Philosophy & Technology.

[CR39] Floridi L, Cowls J, Beltrametti M, Chatila R, Chazerand P, Dignum V (2018). AI4People—An ethical framework for a good AI society: Opportunities, risks, principles, and recommendations. Minds and Machines.

[CR40] Folgieri, R., & Lucchiari, C. (2017). Boosting physical and psychological well-being in rehabilitation through cognitive technologies preliminary results. In *Presented at the 2017 IEEE Canada international humanitarian technology conference (IHTC)* (pp. 75–79).

[CR41] Frank, M. R., Autor, D., Bessen, J. E., Brynjolfsson, E., Cebrian, M., Deming, D. J., & et al. (2019). Toward understanding the impact of artificial intelligence on labor. *Proceedings of the National Academy of Sciences*, *116*(14), 6531–6539 [*]. 10.1073/pnas.1900949116 PMC6452673 30910965

[CR42] Freitas, A., Brito, L., Baras, K., & Silva, J. (2017). Overview of context-sensitive technologies for well-being. In *Presented at the 2017 international conference on internet of things for the global community (IoTGC)* (pp. 1–8).

[CR43] Frey, C. B., & Osborne, M. A. (2017). The future of employment: How susceptible are jobs to computerisation?. *Technological Forecasting and Social Change*, *114*, 254–280 [*].

[CR44] Fröding B, Peterson M (2013). Why computer games can be essential for human flourishing. Journal of Information, Communication and Ethics in Society.

[CR45] Garcia-Ceja E, Osmani V, Mayora O (2016). Automatic stress detection in working environments from smartphones’ accelerometer data: A first step. IEEE Journal of Biomedical and Health Informatics.

[CR46] Giubilini A, Savulescu J (2018). The artificial moral advisor. The “ideal observer” meets artificial intelligence. Philosophy & Technology.

[CR47] Gonzalez, R. (2018). Hey Alexa, what are you doing to my kid’s brain? Wired [Online]. https://www.wired.com/story/hey-alexa-what-are-you-doing-to-my-kids-brain/. Date Accessed November 6, 2018.

[CR48] Gonzalez, R. (2019). Screens might be as bad for mental health as… potatoes. Wired [Online]. https://www.wired.com/story/screens-might-be-as-bad-for-mental-health-as-potatoes/, Date Accessed January 24, 2019.

[CR49] Grubbs JB, Wilt JA, Exline JJ, Pargament KI, Kraus SW (2018). Moral disapproval and perceived addiction to internet pornography: A longitudinal examination. Addiction.

[CR50] Hall JA, Gertz R, Amato J, Pagliari C (2017). Transparency of genetic testing services for “health, wellness and lifestyle”: Analysis of online prepurchase information for UK consumers. European Journal of Human Genetics.

[CR51] Hall, M., Glanz, S., Caton, S., & Weinhardt, C. (2013). Measuring your best you: A gamification framework for well-being measurement. In *Presented at the 2013 international conference on cloud and green computing* (pp. 277–282).

[CR52] Hamm MP, Chisholm A, Shulhan J, Milne A, Scott SD, Given LM, Hartling L (2013). Social media use among patients and caregivers: A scoping review. British Medical Journal Open.

[CR53] Hampshire K, Porter G, Mariwah S, Munthali A, Robson E, Owusu SA (2016). Who bears the cost of “informal mhealth?” Health-workers’ mobile phone practices and associated political-moral economies of care in Ghana and Malawi. Health Policy and Planning.

[CR54] Hart M (2016). Being naked on the internet: Young people’s selfies as intimate edgework. Journal of Youth Studies.

[CR55] Hausman DM (2015). Valuing health: Well-being, freedom, and suffering.

[CR56] Horvitz E, Mulligan D (2015). Data, privacy, and the greater good. Science.

[CR139] Human Genetics Commission. (2010). *A common framework of principles for direct-to-consumer genetic testing services* (pp. 1–15). London: HGC.

[CR57] Huppert FA, So TTC (2013). Flourishing across Europe: Application of a new conceptual framework for defining well-being. Social Indicators Research.

[CR58] IEEE. (2017). *Ethically aligned design, v2*. The IEEE initiative on ethics of autonomous and intelligent systems. https://ethicsinaction.ieee.org. Date Accessed November 22, 2018 [*].

[CR59] Ijsselsteijn, W., de Kort, Y., Midden, C., Eggen, B., & van den Hoven, E. (2006). Persuasive technology for human well-being: Setting the scene (pp. 1–5). In *Presented at the international conference on persuasive technology*. Springer.

[CR60] Jobin A, Ienca M, Vayena E (2019). The global landscape of AI ethics guidelines. Nature Machine Intelligence.

[CR61] Johnson, D., Deterding, S., Kuhn, K., Staneva, A., Stoyanov, S., & Hides, L. (2016). Gamification for health and wellbeing: A systematic review of the literature. *Internet Interventions*, *6*, 89–106 [*]. 10.1016/j.invent.2016.10.002 PMC6096297 30135818

[CR62] Johnson, D., Wyeth, P., & Sweetser, P. (2013). The people-game-play model for understanding videogames’ impact on wellbeing. In *Presented at the 2013 IEEE international games innovation conference (IGIC)* (pp. 85–88).

[CR63] Kahn PH, Gary HE, Shen S (2013). Social and moral relationships with robots: Genetic epistemology in an exponentially increasing technological world. Human Development.

[CR64] Karime, A., Hafidh, B., Khaldi, A., Aljaam, J. M., & Saddik, El, A. (2012). MeMaPads: Enhancing children’s well-being through a physically interactive memory and math games. In *Presented at the 2012 IEEE international instrumentation and measurement technology conference* (pp. 2563–2566).

[CR65] Kartsanis, N., & Murzyn, E. (2016). Me, my game-self, and others: A qualitative exploration of the game-self. In *Presented at the 2016 international conference on interactive technologies and games (ITAG)* (pp. 29–35).

[CR66] Keijzer-Broers, W., Florez-Atehortua, L., & Reuver, M. D. (2016). Prototyping a health and wellbeing platform: An action design research approach. In *Presented at the 2016 49th Hawaii international conference on system sciences* (pp. 3462–3471).

[CR67] Khayal, I. S., & Farid, A. M. (2017). Designing smart cities for citizen health & well-being. In *Presented at the 2017 IEEE first summer school on smart cities (S3C)* (pp. 120–125).

[CR68] Khoury MJ, Ioannidis JPA (2014). Big data meets public health. Science.

[CR69] Khudair, A. A., & Aloshan, M. S. (2015). Caregivers of autistic children: Seeking information in social media. In *Presented at the international conference on information society, i-society 2015*. IEEE (pp. 68–72).

[CR70] Kibel M, Vanstone M (2017). Reconciling ethical and economic conceptions of value in health policy using the capabilities approach: A qualitative investigation of Non-Invasive Prenatal Testing. Social Science and Medicine.

[CR71] Kickbusch I (2006). The health society: The need for a theory. Journal of Epidemiology and Community Health.

[CR72] Klein E, Brown T, Sample M, Truitt AR, Goering S (2015). Engineering the brain: Ethical issues and the introduction of neural devices. Hastings Center Report.

[CR73] Kocielnik, R., Sidorova, N., Maggi, F. M., Ouwerkerk, M., & Westerink, J. H. D. M. (2013). Smart technologies for long-term stress monitoring at work. In *Presented at the proceedings of the 26th IEEE international symposium on computer-based medical systems* (pp. 53–58).

[CR74] Kramer, A. D. I., Guillory, J. E., & Hancock, J. T. (2014). Experimental evidence of massive-scale emotional contagion through social networks. *Proceedings of the National Academy of Sciences of the United States of America*, *111*(24), 8788–8790 [*]. 10.1073/pnas.1320040111 PMC4066473 24889601

[CR75] Krutzinna J (2016). Can a welfarist approach be used to justify a moral duty to cognitively enhance children?. Bioethics.

[CR76] Lehavot K, Ben-Zeev D, Neville RE (2012). Ethical considerations and social media: A case of suicidal postings on facebook. Journal of Dual Diagnosis.

[CR77] Leroy G, Chen H, Rindflesch TC (2014). Smart and connected health. IEEE Intelligent Systems.

[CR78] Levy, N. (2017). Nudges in a post-truth world. *Journal of Medical Ethics*, *43*(8), 495–500 [*]. 10.1136/medethics-2017-104153 PMC5537529 28526778

[CR79] Lyng S (2005). Edgework: The sociology of risk taking.

[CR80] MacKenzie C (2008). Relational autonomy, normative authority and perfectionism. Journal of Social Philosophy.

[CR81] Madary, M., & Metzinger, T. K. (2016). Recommendations for good scientific practice and the consumers of VR-technology. *Frontiers in Robotics and AI*, *3*(3), 1–23 [*].

[CR82] Mahoney DF, Purtilo RB, Webbe FM, Alwan M, Bharucha AJ, Adlam TD (2007). In-home monitoring of persons with dementia: Ethical guidelines for technology research and development. Alzheimer’s & Dementia.

[CR83] Margot-Cattin I, Nygård L (2009). Access technology and dementia care: Influences on residents’ everyday lives in a secure unit. Scandinavian Journal of Occupational Therapy.

[CR84] Markendahl, J., Lundberg, S., Kordas, O., & Movin, S. (2017). On the role and potential of IoT in different industries: Analysis of actor cooperation and challenges for introduction of new technology. In *Presented at the 2017 internet of things business models, users, and networks* (pp. 1–8).

[CR85] Matz, S. C., Kosinski, M., Nave, G., & Stillwell, D. J. (2017). Psychological targeting as an effective approach to digital mass persuasion. *Proceedings of the National Academy of Sciences*, *114*(48), 12714–12719 [*]. 10.1073/pnas.1710966114 PMC5715760 29133409

[CR86] McDaniel BT, Coyne SM, Holmes EK (2012). New mothers and media use: Associations between blogging, social networking, and maternal well-being. Maternal and Child Health Journal.

[CR87] Meyer J, Boll S (2014). Digital health devices for everyone!. IEEE Pervasive Computing.

[CR88] Misselhorn C, Pompe U, Stapleton M (2013). Ethical considerations regarding the use of social robots in the fourth age. GeroPsych.

[CR89] Mitrpanont, J., Sawangphol, W., Chankong, C., Jitsuphap, A., & Wongkhumsin, N. (2018). I-WISH: Integrated well-being IoT system for healthiness. In *Presented at the 2018 15th international joint conference on computer science and software engineering* (pp. 1–6).

[CR90] Mittelstadt B (2017). Designing the health-related internet of things: Ethical principles and guidelines. Information (Switzerland).

[CR91] Mittelstadt B (2017). Ethics of the health-related internet of things: A narrative review. Ethics and Information Technology.

[CR92] Mulvenna, M., McCann, A., O’Kane, M., Henderson, B., Kirby, K., & McCay, D. (2013). A proposed framework for supporting behaviour change by translating personalised activities into measurable benefits. In *Presented at the 2013 international conference on e-business (ICE-B)* (pp. 1–6).

[CR93] Mulvenna, M., Zheng, H., Bond, R., McAllister, P., Wang, H., & Riestra, R. (2017). Participatory design-based requirements elicitation involving people living with dementia towards a home-based platform to monitor emotional wellbeing. In *Presented at the 2017 IEEE international conference on bioinformatics and biomedicine (BIBM)* (pp. 2026–2030).

[CR94] O’Donnell A (2015). Contemplative pedagogy and mindfulness: Developing creative attention in an age of distraction. Journal of Philosophy of Education.

[CR95] Oliveira, Á., Campolargo, M., & Martins, M. (2014). Human smart cities: A human-centric model aiming at the wellbeing and quality of life of citizens. In *Presented at the eChallenges e-2014 conference proceedings* (pp. 1–8).

[CR96] Orben, A., & Przybylski, A. K. (2019). The association between adolescent well-being and digital technology use. *Nature Human Behaviour*, *351*, 1 [*]. 10.1038/s41562-018-0506-1 30944443

[CR97] Palm E (2013). Who cares? Moral obligations in formal and informal care provision in the light of ICT-based home care. Health Care Analysis.

[CR98] Pedaste, M., & Leijen, Ä. (2018). How can advanced technologies support the contemporary learning approach? In *Presented at the 2018 IEEE 18th international conference on advanced learning technologies (ICALT)* (pp. 21–23).

[CR99] Peters, D., Calvo, R. A., & Ryan, R. M. (2018). Designing for motivation, engagement and wellbeing in digital experience. *Frontiers in Psychology*, *9*, 797 [*]. 10.3389/fpsyg.2018.00797 PMC5985470 29892246

[CR100] PEW Research Center. (2018). The future of well-being in a tech-saturated world. http://assets.pewresearch.org/wp–content/uploads/sites/14/2018/04/14154552/PI_2018.04.17_Future-of-Well-Being_FINAL.pdf. Accessed: October 15, 2018 [*].

[CR101] Pot-Kolder R, Geraets C, Veling W, Beilen M, Staring A, Gijsman H, Delespaul A, Gaag M (2018). Virtual-reality-based cognitive behavioural therapy versus waiting list control for paranoid ideation and social avoidance in patients with psychotic disorders: A single-blind randomised controlled trial. The Lancet Psychiatry.

[CR102] Reis, A., Paredes, H., Barroso, I., Monteiro, M. J., Rodrigues, V., Khanal, S. R., & Barroso, J. (2016). Autonomous systems to support social activity of elderly people a prospective approach to a system design. In *Presented at the 2016 1st international conference on technology and innovation in sports, health and wellbeing (TISHW)* (pp. 1–5).

[CR103] Roeser S (2012). Emotional engineers: Toward morally responsible design. Science and Engineering Ethics.

[CR104] Rughiniş C, Rughiniş R, Matei Ş (2015). A touching app voice thinking about ethics of persuasive technology through an analysis of mobile smoking-cessation apps. Ethics and Information Technology.

[CR105] Ryan RM, Deci EL (2000). Self-determination theory and the facilitation of intrinsic motivation, social development, and well-being. American Psychologist.

[CR106] Ryan RM, Deci EL (2017). Self-determination theory.

[CR107] Sánchez, A., Plaza, I., Medrano, C. T., & Garcia-Campayo, J. (2015). Proposal of a mobile application for mindfulness practice in elder people. In *Presented at the IET international conference on technologies for active and assisted living (TechAAL)* (pp. 1–4).

[CR108] Schwab, K. (2017). *The fourth industrial revolution*. London: Penguin [*].

[CR109] Seligman M, Csikszentmihalyi M (2000). Positive psychology: An introduction. American Psychologist.

[CR110] Sen A (2010). The idea of justice.

[CR111] Shahrestani, A., Van Gorp, P., Le Blanc, P., Greidanus, F., de Groot, K., & Leermakers, J. (2017). Unified health gamification can significantly improve well-being in corporate environments. In *Presented at the 2011 annual international conference of the IEEE engineering in medicine and biology society* (pp. 4507–4511). 10.1109/EMBC.2017.8037858 29060899

[CR112] Shahriari, K., & Shahriari, M. (2017). IEEE standard review—Ethically aligned design: A vision for prioritizing human wellbeing with artificial intelligence and autonomous systems. In *Presented at the 2017 IEEE Canada international humanitarian technology conference (IHTC)* (pp. 197–201).

[CR113] Sharkey N, Sharkey A (2012). The eldercare factory. Gerontology.

[CR114] Silva, P. A., Holden, K., & Jordan, P. (2015). Towards a list of heuristics to evaluate smartphone apps targeted at older adults: A study with apps that aim at promoting health and well-being. In *Presented at the 2016 49th Hawaii international conference on system sciences* (pp. 3237–3246).

[CR115] Sinche, S., Barbosa, R., Nunes, D., Figueira, A., & Silva, J. S. (2017). Wireless sensors and mobile phones for human well-being. In *Presented at the 2017 IEEE XXIV international conference on electronics, electrical engineering and computing (INTERCON)* (pp. 1–4).

[CR116] Skinner, B., Leavey, G., & Rothi, D. (2018). Managerialism and teacher professional identity: Impact on well-being among teachers in the UK. *Educational Review*, *00*(00), 1–16 [*].

[CR117] Soraghan, C. J., Boyle, G., Dominguez-Villoria, L., Feighan, J., & Robinson, D. (2015). Challenges of implementing a social prescription service in the clinic: Social prescribing in the LAMP project. In *Presented at the 2015 IEEE international symposium on technology and society (ISTAS)* (pp. 1–6).

[CR118] Spanakis, E. G., Santana, S., Ben-David, B., Marias, K., & Tziraki, C. (2014). Persuasive technology for healthy aging and wellbeing. In *Presented at the 2014 4th international conference on wireless mobile communication and healthcare—transforming healthcare through innovations in mobile and wireless technologies (MOBIHEALTH)* (pp. 23–23).

[CR119] Stiglitz, J., Sen, A., & Fitoussi, J. (2008). Report by the commission on the measurement of economic performance and social progress. https://ec.europa.eu/eurostat/documents/118025/118123/Fitoussi+Commission+report. Date Accessed January 7, 2019.

[CR120] Szablewicz M (2010). The ill effects of “opium for the spirit”: A critical cultural analysis of China’s Internet addiction moral panic. Chinese Journal of Communication.

[CR121] Taddeo M (2014). The struggle between liberties and authorities in the information age. Science and Engineering Ethics.

[CR122] Taddeo, M., & Floridi. L. (2018) How AI can be a force for good. *Science*, 361(6404), 751–752 [*]. 10.1126/science.aat5991 30139858

[CR123] Thieme, A., Wallace, J., Meyer, T. D., & Olivier, P. (2015). Designing for mental wellbeing. In *Presented at the the 2015 British HCI conference* (pp. 1–10). New York, NY: ACM Press.

[CR124] Toboso M (2011). Rethinking disability in Amartya Sen’s approach: ICT and equality of opportunity. Ethics and Information Technology.

[CR125] Tollmar, K., Bentley, F., & Viedma, C. (2012). Mobile health mashups: Making sense of multiple streams of wellbeing and contextual data for presentation on a mobile device. In *Presented at the 2012 6th international conference on pervasive computing technologies for healthcare (PervasiveHealth) and workshops* (pp. 65–72).

[CR126] Toma CL, Hancock JT (2013). Self-affirmation underlies Facebook use. Personality and Social Psychology Bulletin.

[CR127] Twenge, J. M., Martin, G. N., & Campbell, W. K. (2018). Decreases in psychological well-being among American adolescents after 2012 and links to screen time during the rise of smartphone technology. *Emotion*, *18*(6), 765–780 [*]. 10.1037/emo0000403 29355336

[CR128] Tyrväinen, P., Silvennoinen, M., Talvitie-Lamberg, K., Ala-Kitula, A., & Kuoremäki, R. (2018). Identifying opportunities for AI applications in healthcare—Renewing the national healthcare and social services. In *Presented at the 2018 IEEE 6th international conference on serious games and applications for health (SeGAH)* (pp. 1–7).

[CR129] Valkenburg PM, Peter J, Schouten AP (2006). Friend networking sites and their relationship to adolescents’ well-being and social self-esteem. Cyberpsychology, Behavior and Social Networking.

[CR130] Vallée, T., Sedki, K., Despres, S., Jaulant, M., Tabia, K., & Ugon, A. (2016). On personalization in IoT. In *Presented at the 2016 international conference on computational science and computational intelligence (CSCI)* (pp. 186–191).

[CR131] Vallor S (2010). Social networking technology and the virtues. Ethics and Information Technology.

[CR132] Vallor, S. (2016). *Technology and the virtues.* Oxford: Oxford University Press [*].

[CR133] Van Hooren RH, Van Den Borne BW, Curfs LMG, Widdershoven GAM (2007). Ethics of prevention: An interactive computer-tailored program. Scandinavian Journal of Public Health.

[CR134] Verduyn P, Lee DS, Park J, Shablack H, Orvell A, Bayer J (2015). Passive facebook usage undermines affective well-being: Experimental and longitudinal evidence. Journal of Experimental Psychology: General.

[CR135] Vodafone Institute for Society and Communications. (2018). *The Tech Devide*. https://www.vodafone-institut.de/wp-content/uploads/2018/10/The-Tech-Divide-People-and-Society.pdf, Date Accessed: January 7, 2019 [*].

[CR136] Weiss, G. M., Lockhart, J. W., Pulickal, T. T., McHugh, P. T., Ronan, I. H., & Timko, J. L. (2016). Actitracker: A smartphone-based activity recognition system for improving health and well-being. In *Presented at the 2016 IEEE international conference on data science and advanced analytics (DSAA)* (pp. 682–688).

[CR137] World Economic Forum. (2018). The future of jobs report 2018. http://www3.weforum.org/docs/WEF_Future_of_Jobs_2018.pdf, Date accessed November 10, 2018 [*].

[CR138] World Health Organisation. (2005). WHO eHealth Resolution. https://www.who.int/healthacademy/news/en/, Date Accessed: January 24, 2019.

